# Innovations and developments in single cell protein: Bibliometric review and patents analysis

**DOI:** 10.3389/fmicb.2022.1093464

**Published:** 2023-01-13

**Authors:** Gislane Oliveira Ribeiro, Leticia de Alencar Pereira Rodrigues, Thiale Borges Silva dos Santos, João Pedro Santos Alves, Roseane Santos Oliveira, Tatiana Barreto Rocha Nery, Josiane Dantas Viana Barbosa, Milena Botelho Pereira Soares

**Affiliations:** ^1^Biotechnology Laboratory, Alternative Protein Competence Center, University Center SENAI CIMATEC, Salvador, Brazil; ^2^SENAI Institute of Innovation (ISI) in Health Advanced Systems (CIMATEC ISI SAS), University Center SENAI/CIMATEC, Salvador, Bahia, Brazil; ^3^Gonçalo Moniz Institute, FIOCRUZ, Salvador, Bahia, Brazil

**Keywords:** alternative protein, bibliometric analysis, fermentation, meat analogues, prospecting, patents

## Abstract

**Background:**

Global demand for food products derived from alternative proteins and produced through sustainable technological routes is increasing. Evaluation of research progress, main trends and developments in the field are valuable to identify evolutionary nuances.

**Methods:**

In this study, a bibliometric analysis and search of patents on alternative proteins from fermentation processes was carried out using the Web of Science and Derwent World Patents Index^™^ databases, using the keywords and Boolean operators “fermentation” AND “single cell protein” OR “single-cell protein.” The dataset was processed and graphics generated using the bibliometric software VOSviewer and OriginPro 8.1.

**Results:**

The analysis performed recovered a total of 360 articles, of which 271 were research articles, 49 literature review articles and 40 publications distributed in different categories, such as reprint, proceedings paper, meeting abstract among others. In addition, 397 patents related to the field were identified, with China being the country with the largest number of publications and patents deposits. While this topic is largely interdisciplinary, the majority of work is in the area of Biotechnology Applied Microbiology, which boasts the largest number of publications. The area with the most patent filings is the food sector, with particular emphasis on the fields of biochemistry, beverages, microbiology, enzymology and genetic engineering. Among these patents, 110 are active, with industries or companies being the largest depositors. Keyword analysis revealed that the area of study involving single cell protein has included investigation into types of microorganisms, fermentation, and substrates (showing a strong trend in the use of agro-industrial by-products) as well as optimization of production processes.

**Conclusion:**

This bibliometric analysis provided important information, challenges, and trends on this relevant subject.

## 1. Introduction

Proteins are essential nutrients in the human diet and play important roles in the body. The composition of the constituent amino acids of a protein relay its nutritional value (Bratosin et al., 2021), and foods such as meat, milk and eggs are notable for having proteins of high biological value, providing all essential amino acids (Greene and McAdam, 1983). Throughout the past several decades, the search for proteins has increased notably in the setting of increased utilization. Global consumption of animal proteins reached 327,683 kt in the triennium 2019–2021, with a market whose value is expected to reach 7.3 trillion dollars by 2025 (Bohrer, 2019; OECD/FAO, 2022). Razzaq et al. (2022) also describe an estimated increase in the demand for proteins of 50% and for products based on animal protein of 102% by the year 2050.

The growing demand for this nutritional source stems from population growth and is driven by socioeconomic changes such as increasing urbanization and rising incomes. However, large-scale production of animal-derived proteins is reported to be one of the main drivers of biodiversity loss, climate change and freshwater depletion (Munialo et al., 2022). For this reason, studies have been carried out with increased focus on alternative sources of proteins and methods that aim to prioritize animal welfare and sustainability. The great challenge, however, is to develop analogues of animal-derived products that have nutritional and sensory attributes similar to those of traditional meat (Ahmad et al., 2022).

Available alternatives to animal protein currently include analogues from plant sources (Chiang et al., 2019; de Angelis et al., 2020), insect proteins (Azagoh et al., 2016; Chatsuwan et al., 2018), cultured meats (Hanga et al., 2020; Shahin-Shamsabadi and Selvaganapathy, 2022) and unicellular protein (SCP; Aggelopoulos et al., 2014; Ahmad et al., 2022). SCP is an interesting alternative due to its nutritional profile, as it contains proteins of high biological value (11.25%), including all essential amino acids, dietary fibers (6%), minerals (iron, copper, zinc, selenium, manganese and phosphorus) and vitamins (riboflavin, folic acid, biotin, ascorbic acid and vitamin D; Bratosin et al., 2021; Ahmad et al., 2022). Additionally, the protein biomass is composed of a tangled conglomeration of mycoprotein hyphae that results in a meat-like texture and lends to sensory properties similar to meat (Ahmad et al., 2022).

SCPs are obtained from the microbial biomass of bacteria, fungi and microalgae. The production of alternative proteins through this technology has been developed over the last 100 years (Reihani and Khosravi-Darani, 2019), with applications in human and animal nutrition. However, increased interest in the field has been aroused in recent years, with the advent of new technologies such as the use of different microbial species, nutrient enhancement and genetic engineering which can effectively increase biomass production (Wikandari et al., 2021). Currently, million-dollar investments have been made in this sector, such as that of the British company Deep Branch Biotechnology, which invested 2.5 million euros in 2020, for the development of fermentation platforms for SCPs (Linden, 2022). SCP is a technology used in a variety of industrial sectors, and as such remains a topic whose importance continues to increase. Thus, identifying main trends and developments in the field through this bibliometric study and patent survey is both relevant and quite interesting.

The surveying of patents is important to assess technological progress and the diffusion of knowledge. Bibliometric analysis, in turn, is a tool widely used to present perspectives in various fields of research. In addition, it aims to analyze publications and patents, citations and their results, helping researchers choose fields of interest or identify possible collaborators (Gabriel da Rosa et al., 2022). Since such a collection of information optimizes scientific research and exposes knowledge gaps, it allows for better understanding and improvement of the research area. This study aimed to carry out a bibliometric analysis on the production of alternative proteins from fermentation processes by describing the current landscape of publications and patents related to the topic.

## 2. Materials and methods

**Figure 1:**
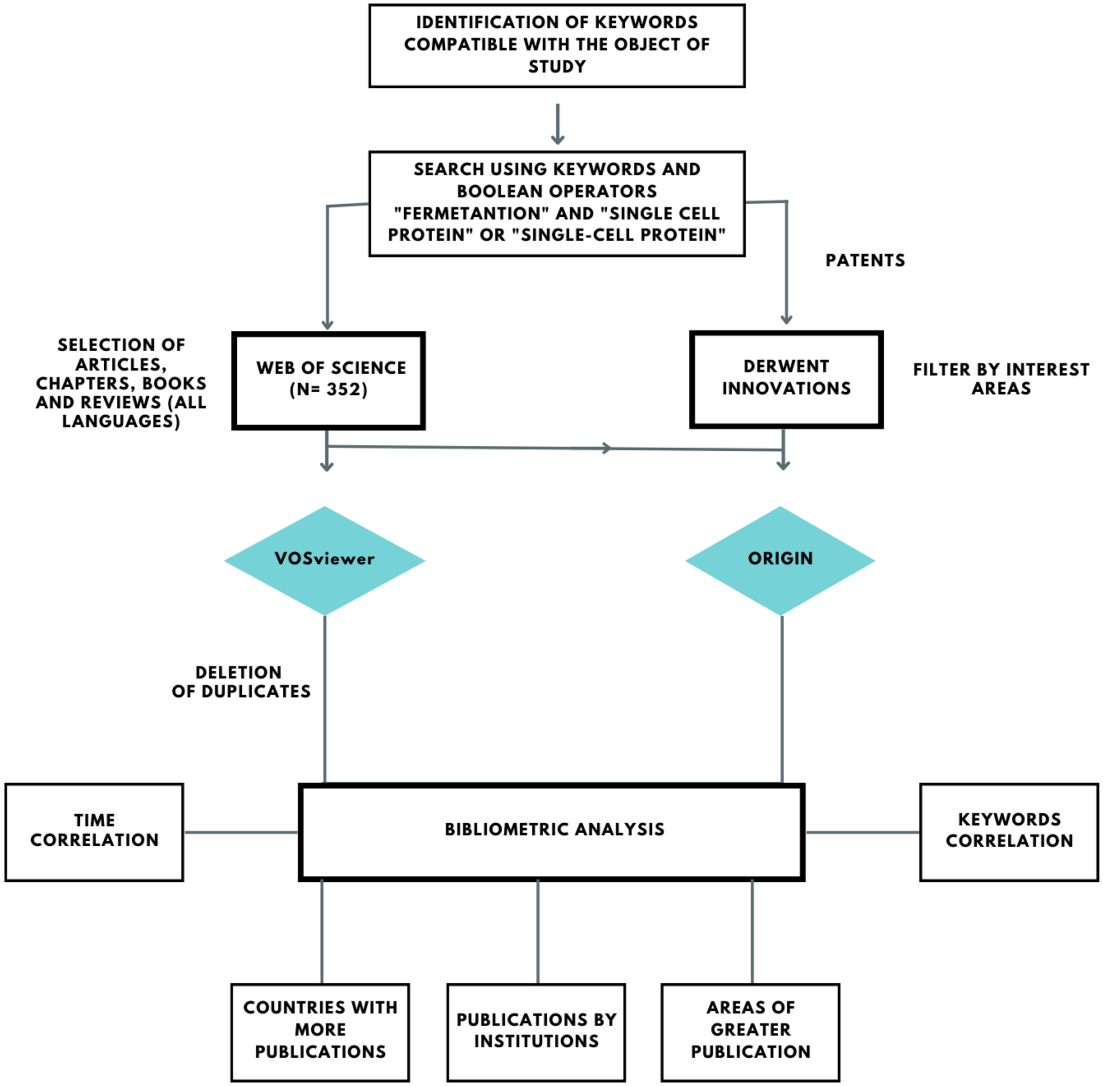
Methodological steps performed in the present study for the bibliometric search and analysis.

For the bibliometric study, data collection was carried out in August 2022 and utilized scientific publications indexed in the Web of Science (WoS) database and patents registered in the Derwent World Patents Index™ (DWPI). DWPI is one of the most robust databases available today and contains patent applications and grants sourced by 44 patent-issuing authorities from 90 countries and organizations. The search was carried out in the “advanced search” section of the WoS platform, using the combination of keywords and Boolean operators “fermentation” AND “single cell protein” OR “single-cell protein.” Through this query, it was possible to filter all publications based on the selected words included in the title, abstract and keywords, avoiding incompatibilities by excluding words present only in the references which were not the objective of this study. No timeframe limitations were applied to the parameters of this search in order that the entire period of publications was covered and the technique of interest could be analyzed chronologically. After refining and defining documents relevant to the scope of this prospective study, those works deemed significant were utilized to illustrate the trends of interest. The information was exported from the platforms and the graphics were generated using the OriginPro 8.1 software. Then, the publications resulting from the WoS search were analyzed and the trends were based on the recurrence of keywords using VOSviewer software to build maps of correlations of keywords and journals. Figure 1 presents a methodological scheme used in the bibliographic research.

## 3. Results

### 3.1. Evolution of publications, patent filings and research areas

The data described in this study consist of findings from WoS and DWPI platform searches using the terms “single cell protein” or “single-cell protein” and “fermentation.” The term “fermentation” was used because the subject of this study are the proteins obtained by the fermentation process, and the keyword “single cell protein” was defined because it is the most used term in studies in the field. According to Mateles et al. (1967) initially the terms used for proteins obtained by fermentation were “microbial protein” and “petroprotein,” which were replaced in 1975 by single cell protein. The term “single cell protein” refers to dried microbial cells or whole proteins extracted from microbial cell cultures. It can also be called biomass, bioprotein or microbial protein (Akalya et al., 2017).

**Figure 2:**
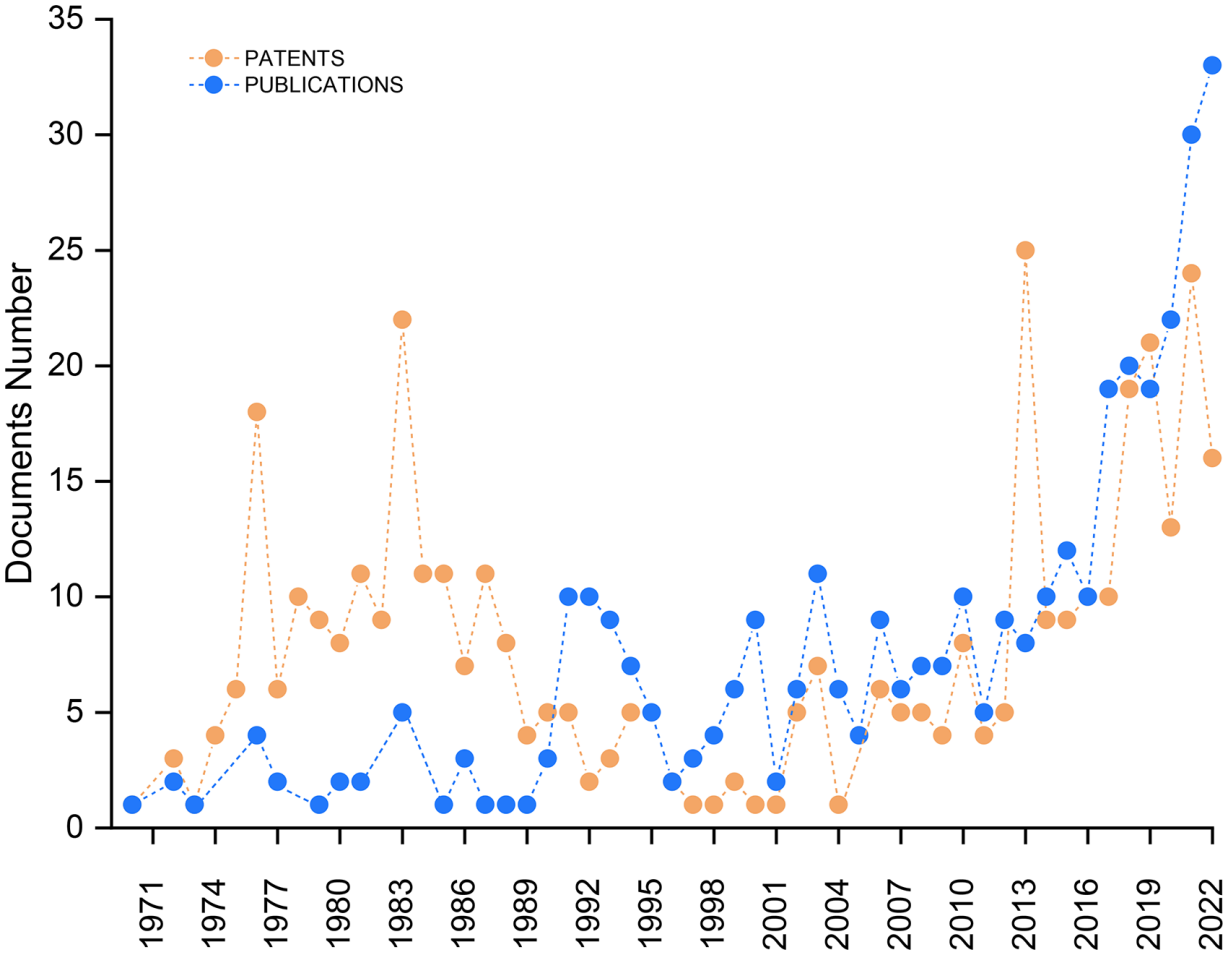
Evolution of publications and patent filings in the period 1970–2022.

The evolution of research related to SCP can be seen in Figure 2. The literature search carried out through the WoS database revealed 360 publications, including 271 research articles, 49 literature review articles and 40 publications distributed in different categories, such as reprint, proceedings paper, meeting abstract among others. The first finding dates back to 1970 with a work by Cama and Edwards (1970), who proposed the production of single cell protein from the growth of *Candida utilis* using sodium acetate, a residual inorganic compound generated in the industrial production of acetic acid and hydroxyethylcellulose, as a carbon source. For two decades there was an average of 1.4 publications per year. However, from 1990 to 2010, this area of research evolved, with an average of 6 publications per year. An increased interest in the field has been observed in the last decade, reflected by an impressive number of publications including 20 in the year 2017 and a 65% increase in the number of publications from 2020 to 2021. Furthermore, an additional increase is anticipated for this year (2022), in which 33 documents had already been published as of the month of august.

Exponential increase in the volume of publications contributing to this area is related to new global trends which involve several dimensions of sustainability, including economic, social, environmental, health and animal welfare issues. Although the specific reasons for increased interest in alternative proteins are multifaceted and may differ between countries and cultures, several studies have attributed this finding to the growing concern with health, animal welfare and the environment on the part of consumers (Parlasca and Qaim, 2022).

Regarding the number of patents (Figure 2), in the period from 1970 to 2022, a total of 397 deposits were found. Searches related to this topic were performed on the DWPI platform using the same Boolean terms and operators used in the search for article publications, and findings of interest were manually filtered. After refining the data, patents were organized according to the year of filing. The oldest patent found was filed in 1970 by the Chinese Petroleum Corporation Taipei, which patented the use of a strain of *Pseudomonas* (N° 5742, ATCC 21174) for the production of a dough with 60–80% protein using hydrocarbons (natural gas) or carbohydrates (glucose or molasses) as substrate. By observing the evolution of trends in this area over the years, it is notable that the number of patent filings was significantly higher than the number of article publications throughout the 20^th^ century. In one decade in particular, from 1970 to 1980, there was an impressive increase in patent filings which peaked in 1976 at 18. Of contributing nations during this period, the United States filed the majority of these patents, or 10 of the total18.

In 1983, a new peak was observed in the number of patent registrations with 22 filings, 9 of which were from East Germany. Another exponential increase can be seen from the year 2000 onward, with a sharp peak in 2013 composed of 25 total patents, 23 of which were the contributions of China. This growing trend has been accentuated in recent years, showing the positive influence of patenting on the development of companies. Blind et al. (2022) describe that patents are increasingly used for strategic reasons, stimulating not only the development of products, but also hindering competitors and securing valuable time in the commercialization of products.

**Figure 3:**
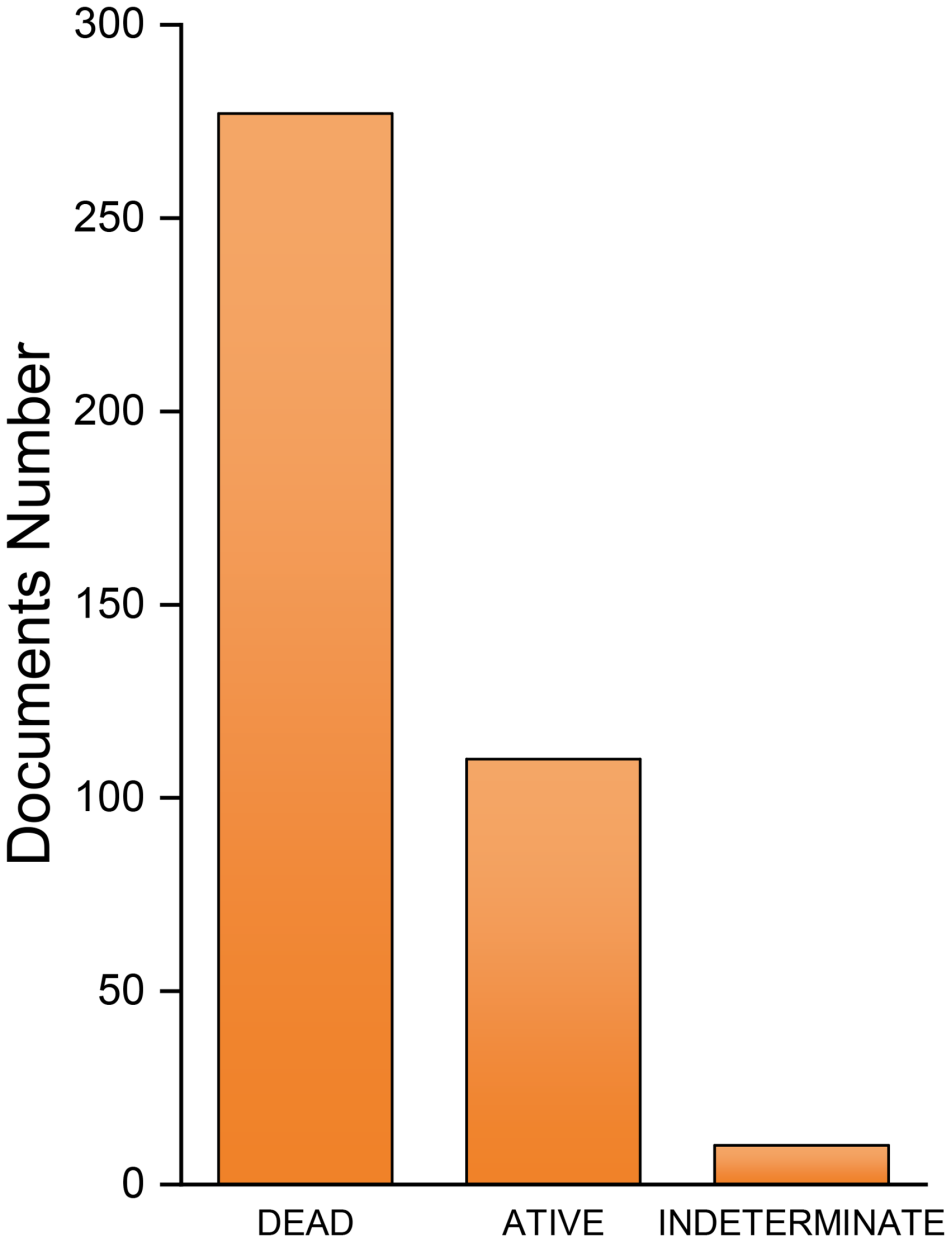
Current status of patents with keywords “fermentation” and “single cell protein” or “single-cell protein” extracted from Derwent World Patents Index^™^.

Among the patents found, 277 have expired, 110 are active and 10 are in indeterminate status (Figure 3). In regard to inactive deposits, special emphasis is given to the patent for the Quorn brand by Marlow Foods. This patent has expired and Quorn products continue to be marketed. Quorn is the best example of a well-known mycoprotein product, which is marketed in all European Union countries, as well as in the United States, Australia, New Zealand, Canada and, more recently, in Asian markets. Seven Quorn products have been introduced as chilled or frozen foods, with an estimated 5 billion servings consumed worldwide since launch. The mycoprotein market in the United States is estimated to represent almost US$ 149.6 million in 2020 and currently, the mycoproteins produced by Quorn are estimated at 25.000 ton DM/year, with a global market of around 214 million euros (Finnigan et al., 2017; Hashempour-Baltork et al., 2020; Ahmad et al., 2022).

Among active patents, the 5 most recent are listed in Table 1, which highlights three modalities in which patents are submitted in the food sector: production process, raw material, and genetic engineering. Three of these submissions were submitted by the Tianjin Institute of Industrial Biotechnology Chinese Academy of Sciences, highlighting Chinese advances and investments in innovation in the food industry. Patent number WO2022008478A2 refers to the modification of the single cell protein production process with the use of a loop reactor in the initial fermentation process and an innocuous one comprising one or more methanogenic microorganisms. On the other hand, patent number CN114107073A refers to the use of molasses as a culture medium for the filamentous fungus *Fusarium venenatum* in the production of food, health products, or feed, and patent number WO2022038588A2 investigates changes in pH for better yield of *Lactobacillus* for the production of single cell protein. The following two patents highlight the potential for genetic engineering in this area. Patent number CN114214229A refers to the method of production of *Pantococcus pantotrophus* by denitrification and property of the new strain named MA3, while patent number CN114410486A is related to genetic modification in *Aspergillus oryzae* to improve biomass degradation. Techniques of this nature, which use genetic engineering with the aim, for example, of inducing the expression of animal proteins in microorganisms or increasing their production and proliferation capacity, have been well documented, reflected in the number of publications and patent filings.

**Table 1:** Patents on single cell protein submitted in 2022.

DWPI Family	Title	Year	Assignee/Applicant	Country
WO2022008478A2	Process for producing single cell protein | processus de production de protéine unicellulaire	2022	UNIBIO A/S	EUROPEAN PATENT OFFICE (EPO)
CN114107073A	A method for producing hypha protein by using molasses	2022	Tianjin Institute of Industrial Biotechnology Chinese Academy of Sciences	China
WO2022038588A2	Method for simultaneously producing single cell protein and high protein feed from corn fermentation mash	2022	SDIC Biotechnology Investment Co. Ltd.	NOVOZYMES A/S, DK
CN114214229A	Pantococcus pantotrophus strain MA3, production method and application thereof	2022	Tianjin Institute of Industrial Biotechnology Chinese Academy of Sciences	China
CN114410486A	Aspergillus oryzae strain and application thereof in development of feed protein	2022	Tianjin Institute of Industrial Biotechnology Chinese Academy of Sciences	China

Data extracted from the Derwent World Patents Index^™^.

The multidisciplinary nature of SCP is exemplified by the variety of sources for patent submission identified through the International Patent Classification (IPC; Table 2). Deposits arose from a number of different areas, including 387 deposits related to the food sector. This sector, in particular, contributes to the highest percentage of patents registered with the IPC C12 and includes technologies whose application of microorganisms and fermentation is customary and well-established, such as processes of beer, alcohol, wine and vinegar production.

**Table 2:** Percentage of patents found considering the 10 main class IPCs registered and definition of the corresponding IPCs.

**IPC Class**	**% IPC**	**Definition**
**C12**	39.9%	Biochemistry; beer; spirits; wine; vinegar; microbiology; enzymology; mutation or genetic engineering
**A23**	28.5%	Foods or foodstuffs; treatment thereof, not covered by other classes
**C02**	4.3%	Treatment of water, waste water, sewage, or sludge
**A01**	3.9%	Agriculture; forestry; animal husbandry; hunting; trapping; fishing
**C07**	3.3%	Organic chemistry
**B01**	3.5%	Physical or chemical processes or apparatus in general
**C10**	2.6%	Petroleum, gas or coke industries; technical gases containing carbon monoxide; fuels; lubricants; peat
**C05**	2.4%	Fertilisers; manufacture thereof
**G01**	2.2%	Measurement; test

Data extracted from Derwent World Patents Index^™^ and meaning of IPCs obtained from WIPO (World Intellectual Property Organization).

Considering publications related to SCP, it is obvious that a number of different sectors have been involved in this research. Food science and technology, energy, chemistry, agricultural engineering, environmental sciences, agronomy, biology, ecology, and economics make up a sample of these involved fields, further highlighting how interesting and multidisciplinary this subject is. According to the analysis extracted from WoS, the four main areas interested in this technology are Biotechnology Applied Microbiology (46%), Food Science Technology (15.9%), Energy Fuels (15%) and Engineering Chemical (10.8%).

### 3.2. Bibliometric study of countries, institutions and journals

**Figure 4:**
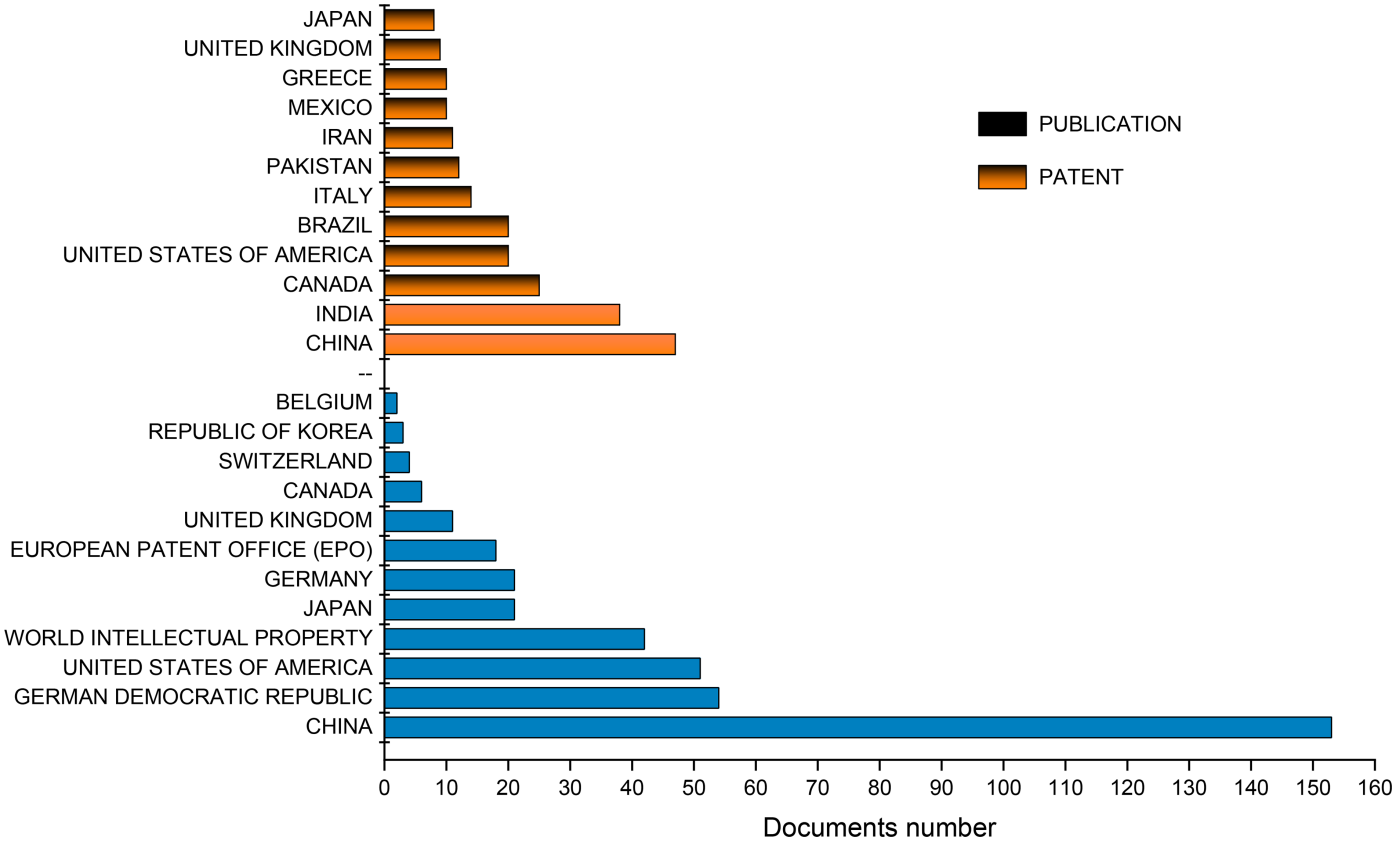
Main countries that most published articles and filed patents using the terms “fermentation” and “single cell protein” or “single-cell protein.”

Research on SCP has been performed in different regions of the world, with China (13%), India (10%), Canada (7%), Brazil (6%) and the United States (6%) being the top five countries with the largest number of publications in the area (Figure 4). Regarding patents, the main applicant countries are China (38%), East Germany (14%), the United States (13%), World Intellectual Property (11%) and Japan (5%; Figure 4). The country with the largest number of publications and patents is China, with several industries and active educational institutions. According to Ritala et al. (2017), China held 70% of patent filings in the period from 2001 to 2016. The main emphasis of their projects revolved around the production of SCP by fermentation of food and agricultural waste using bacteria, yeasts and mixed populations, being the production of SCP often combined with bioremediation.

Currently, in addition to international funding and initiatives, China is also home to the Lever China Alternative Protein Fund. Created by the Chinese company Lever Foods, this fund provides consulting services to leading national and international food companies, startups and investors. Lever Foods aims to help companies gain acceptance for the production of alternative proteins, promote collaboration between institutions and identify investment opportunities with the greatest potential. In addition, the Protein Fund aims to support and incentivize the production of alternative proteins in China by entrepreneurs and early stage companies (Lever Foods, 2022).

**Figure 5:**
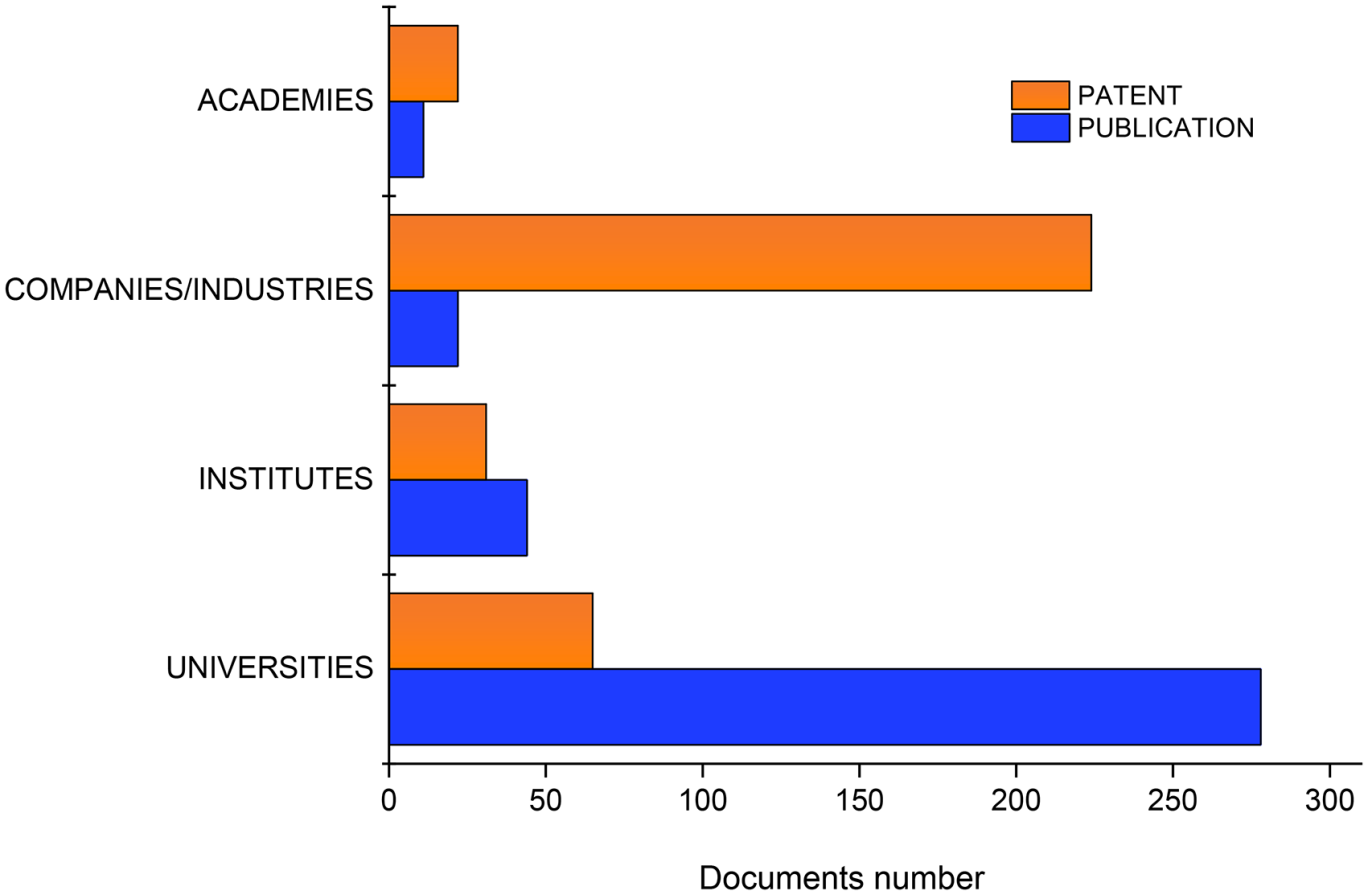
Number of patent filings and publications submitted by different institutions.

As for patent filing organizations, it was observed that 58% of all filings were submitted by industries or companies, and only 16% were filed by universities (Figure 5). According to Blind et al. (2022), the most prominent route of knowledge dissemination by companies is patenting, which allows them to appropriate the returns from their innovations through exclusive and time-restricted rights.

As for publications, the predominant contributing entity is universities (77%; Figure 5) which together with institutes and academies are establishments focused on scientific development. However, a potential challenge for these entities is that patent are not necessarily prioritized, and therefore, the knowledge represented in their publications is not always reflected in patent submissions. Industries and companies, on the other hand, seek commercial guarantees in the filing of patents, or harbor knowledge through industrial secrecy. Among such companies, Phillips Petroleum Corporation stands out with the highest number of patent filings. Whereas, the Tianjin Institute of Industrial Biotechnology Chinese Academy of Sciences among the institutes, the AKAD WISSENSCHAFTEN DDR among the academies and among the universities to Nanjing Tech University. It is important to note that the company Phillips Petroleum Corporation, in addition to having patent deposits, was also identified with 6% of publications.

Given the strong performance of international initiatives that encourage the development of alternative food sources, an even greater increase in the number of patents and publications is expected. Among these initiatives, the Good Food Institute (GFI) has been specifically encouraging the development and production of alternative protein sources. One of the technologies promoted by the GFI is the production of meat analogues from single cell protein. In addition to funding notices, the GFI encourages collaboration between research groups and companies. Recently, the GFI listed companies whose technological focus is the production of animal-free meat through the fermentation process for food production.

Table 3 lists some companies that use single cell protein technology for food production. As mentioned earlier, this technology is exploited by several countries. Because this is not a new technology, companies like Bega Cheese Ltd. have been using it for decades. That is not to say, however, that novel approaches aren’t being attempted in the use of single cell protein. For example, the company Those Vegan Cowboys founded in 2020 has had success in using genetic recombination to produce vegan cheese.

**Table 3:** Companies that currently use single cell protein technology for food production.

**Company**	**Founded year**	**Country**	**Technology/Microorganism**
Kernel mycofood	2019	Argentina	Decentralized production of mycoprotein.
Bega cheese Ltd.	1899	Australia	*Saccharomyces*.
Those vegan cowboys	2020	Belgium	Production of recombinant casein to make vegan cheese (from the founders of The Vegetarian Butcher).
Lesaffreb	1853	Brazil	Yeast.
lallemand Inc.	1952	Canadá	Yeast and bacteria.
cbh Qingdao Co., Ltd.	2002	China	Bacterial fermentation (n.a.).
Novacca	2018	Denmark	Milk proteins using fermentation platform
Solar foods	2017	Finland	Electrolysis-enabled novel protein under Solein brand for food ingredients, plant-based meat, and cultivated meat
Aquacultured foods	2020	France	Whole-cut seafood analogues.
Bluebio Tech Int.	2000	Germany	*Spirulina*, *Chlorella* sp.
Foods myco mizoram	2019	India	Mycelium-derived meat products
Chunk	2020	Israel	Whole-cut meat analogues processed with solid-state fermentation
Kinoko-Tech	2019	Israel	Mycelium-derived meat products
Pura	2019	Italy	Mycoprotein production and fermentation to enhance plant-based foods
The protein brewery	2019	Netherlands	Fungi-based protein to replace meat under the Fermotein brand and fungi-produced egg proteins
Mycovation	2020	Singapore	Meat analogues from mycelium
Mycorena	2017	Sweden	Fungi-based protein for food applications (Swedish meatballs) using industrial side streams
Cultivated	2020	Switzerland	Microbial fermentation to develop alternatives to dairy products
Cyanotech	1983	United States	*Spirulina platensis*
Marlow foods Ltd.	1969	United States	*Fusarium venenatum*
Phillips petroleum company	1917	United States	*Pichia* sp., *Torula* sp.

**Figure 6:**
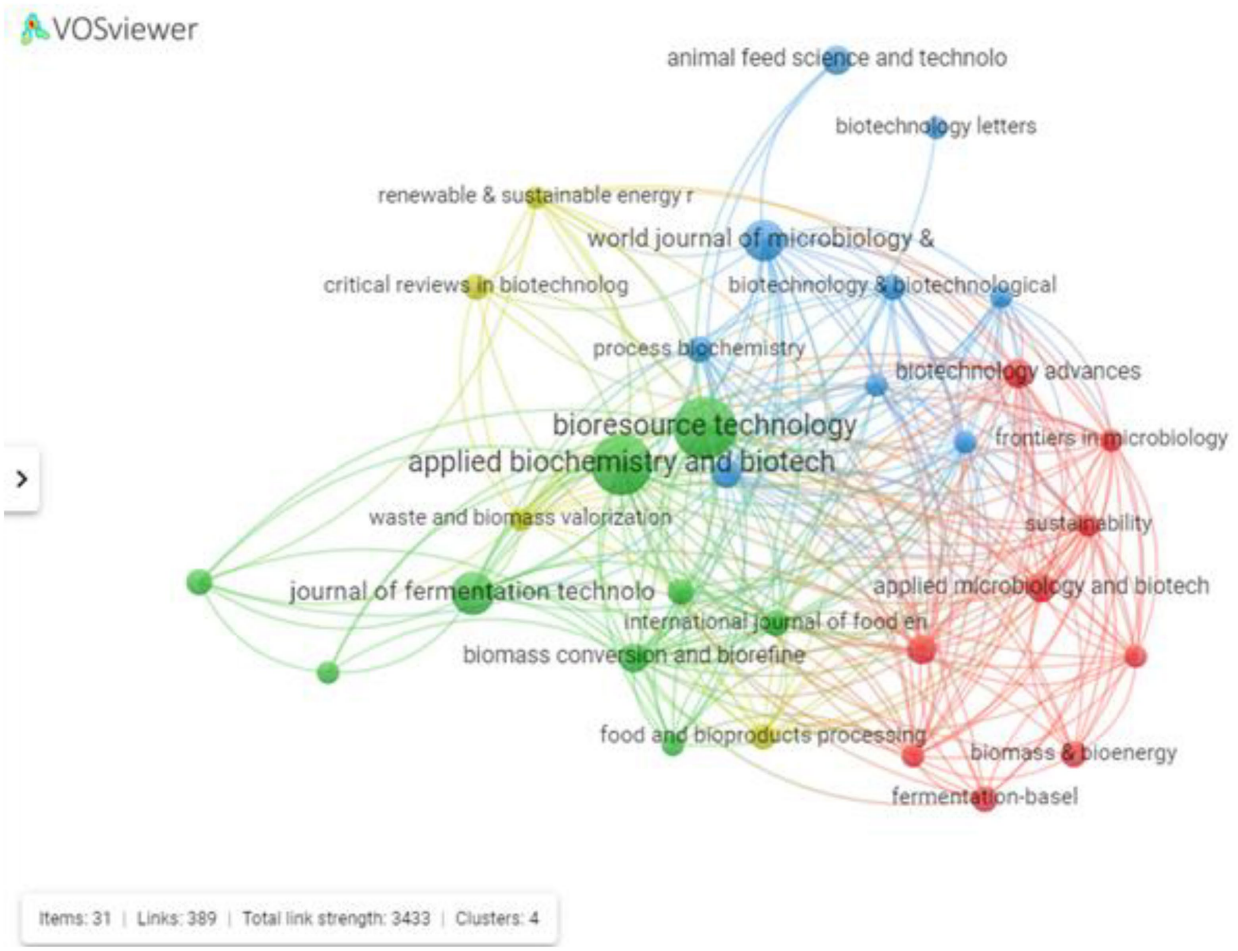
Network map with the main journals with publications using the keywords and Boolean operators “fermentation” and “single cell protein” or “single-cell protein.”

The literature search performed through the WoS database revealed that articles on SCP were published in journals representative of a number of different scientific areas. A total of 199 journals were identified, of which only 10 have at least five publications in the area. The periodicals *Bioresource Technology*, *Applied Biochemistry and Biotechnology*, *Journal of Fermentation Technology* and *World Journal of Microbiology Biotechnology* are the journals with the largest number of publications. *Frontiers in Microbiology* has 3 recent publications related to this area, 2 of which are among the 10 most cited works. Its publications address relevant topics, such as the research by Su et al. (2021) who worked with the fermentation of agro-industrial residues to improve the nutritional value and bioavailability of nutrients to be used as animal feed and the literature review by Ritala et al. (2017) who presented the advances in SCP production from various organisms, giving an overview of commercial exploitation, a review of advances in the patent landscape (2001–2016) and a list of industrial players (Figure 6).

### 3.3. Study of the main keywords in the area of single cell protein

**Figure 7:**
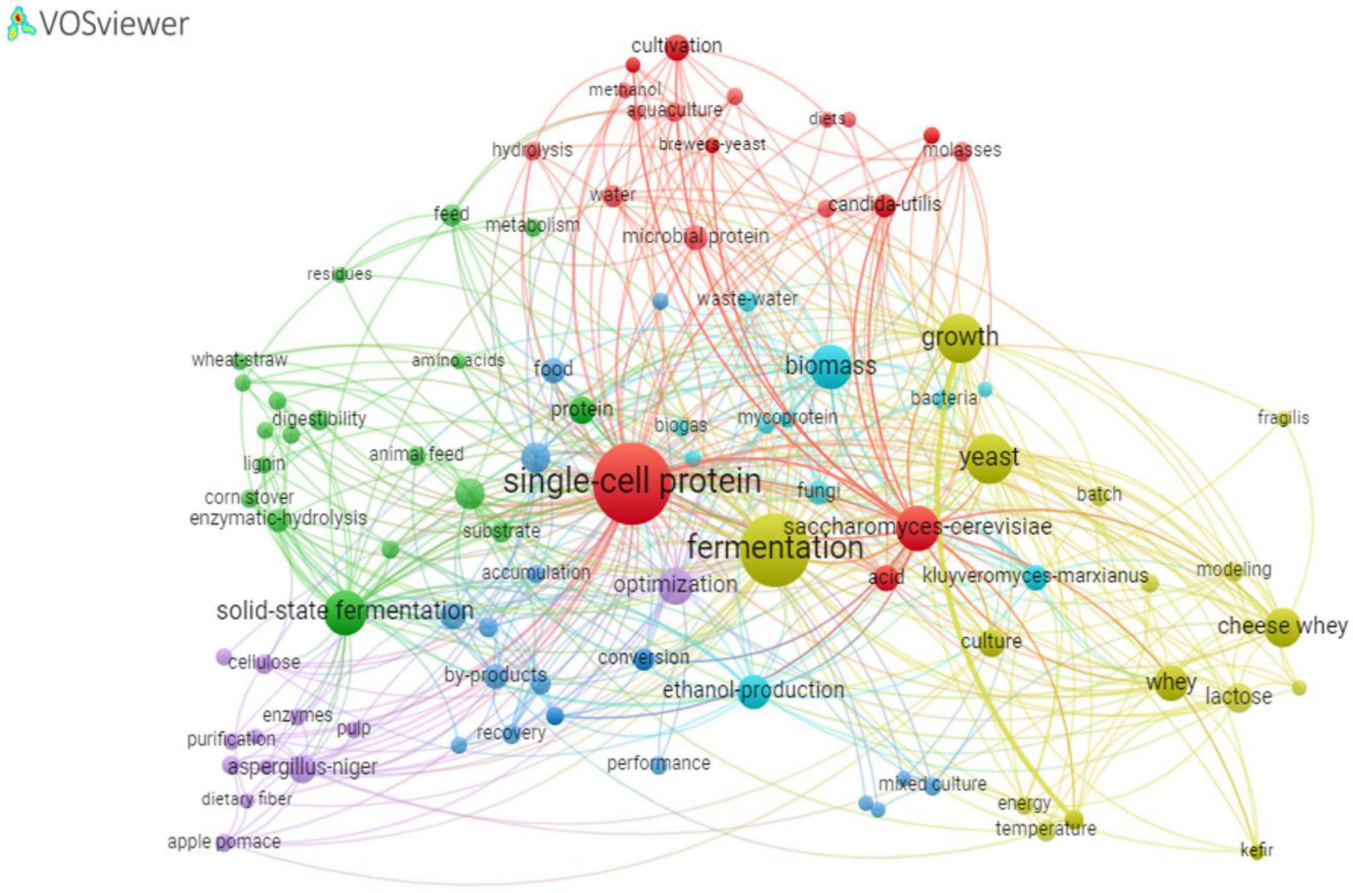
Network map showing correlations between the most used keywords in the articles based on the keywords and Boolean operators “fermentation” and “single cell protein” or “single cell protein.”

Publications involving single cell protein comprise several areas of knowledge as demonstrated above. Thus, a search for the predominant keywords is imperative to determine trends in emerging themes and identify critical points that may be of interest as areas of research, development and innovation (Barbosa et al., 2022). The analysis of keywords related to SCP, using the VOSwiewer program, produced 1701 results, where only 157 responses reached the limit of 5 co-occurrences. The resultant network map depicts 6 clusters, where the keyword, “single cell protein” correlates with all of them (Figure 7). The most cited keywords are in reference to themes of microorganisms, types of fermentation, types of substrates and process optimization. The words: fermentation, yeast, cultivation, biomass, *Sacharomyces cerevisae*, cheese whey, optimization and bioconversion are the most frequently identified.

Different microorganisms can be used for the production of SCP, such as bacteria, algae and fungi as shown in Table 4. From the data extracted from WoS, fungi were identified as the predominant microorganisms, representing 84% of the species cited in the studies, whether microorganisms applied individually or in consortium, with yeasts and filamentous fungi being the predominant microorganisms. At a lower percentage are bacteria, followed by microalgae. The microorganism species that presented the highest occurrence was *Saccharomyces cerevisae*, as can be seen in the map of correlations (Figure 7). According to Parapouli et al. (2020), this yeast is widely used in various biotechnological applications, some of which date back several millennia. In the food sector, it is used for the production of breads and fermented beverages and has been widely used in the production of biofuels, such as second-generation bioethanol (Sjöde et al., 2007; Akter et al., 2020; Kamal et al., 2022).

**Table 4:** Microorganisms used in the production of SCP, type of fermentation, and substrates.

**Microrganism**	**Type of fermentation**	**Substrate**	**Author/Reference**
**Yeast**			
*Yarrowia lipolytica*	Submerged fermentation	Inulin and artichoke flour	(Cui et al., 2011)
*Saccaromyces cerevisiae, Rhodospeudomonas, Trichoderma harzianum*	Submerged fermentation	Pineapple waste	(Akalya et al., 2017)
*Saccharomyces cerevisiae*	Submerged fermentation	Blend of Apples, Pears, Bananas, Strawberries, Cauliflower, Zucchini, Pepper and Molasses.	(Gervasi et al., 2018)
*Candida utilis* ATCC 9950	Submerged fermentation	Potato waste water	(Kurcz et al., 2018)
*Saccharomyces cerevisiae* , *Kluyveromyces marxianus*	Solid state fermentation	Cheese whey and molasses	(Aggelopoulos et al., 2014)
*Hanseniaspora uvarum KKUY-0084 and Zygosaccharomyces rouxii KKUY-0157*	Submerged fermentation	Juice of spoiled dates	(Hashem et al., 2014)
**Filamentous fungi**			
*Neurospora intermedia*	Solid state fermentation	Wheat and whole meal bread waste	(Gmoser et al., 2019)
*Paradendryphiela salina* 100,654	Submerged fermentation	*Ulva* spp.	(Salgado et al., 2021)
*Paradendryphiella salina 100,654*	Submerged fermentation	*Macrocystis pyrifera*	(Salgado et al., 2021)
*Pleurotus florida*	Solid state fermentation	Straw of wheat	(Ahmadi et al., 2010)
*Kluyveromyces marxianus*	Submerged fermentation	Cheese whey	(Yadav et al., 2014)
*Pleurotus sapidus*	Submerged fermentation	Apple pomace	(Ahlborn et al., 2019)
*Aspergillus Niger*	Submerged fermentation	Orange, pineapple, banana, watermelon and cucumber waste	(Oshoma and Eguakun-Owie, 2018)
*Fusarium venenatum*	Solid state fermentation	Cane and brown sugar	(Thomas et al., 2017)
*Fusarium venenatum ATCC 20334*	Submerged fermentation	Dates Juice	(Hosseini and Khosravi-Darani, 2011)
*Fusarium venenatum* IR372C	Submerged fermentation	Dates	(Hashempour-Baltork et al., 2020)
**Bacteria**			
*Bacterium Rhodopseudomonas faecalis PA2*	Submerged fermentation	Domestic wastewater	(Saejung and Ampornpat, 2019)
*Bacillus pumilus*	Fermentation	Potato starch waste	(Li et al., 2013)
*Bacillus sp. (KISRI TMIA, NCIMB 40040*	Submerged fermentation	Culture medium	(Banat et al., 1992)
*Bacillus subtilis NRRL NRS-744, Bacillus cereus NRRL B-3711, Escherichia coli*	Submerged fermentation	Ram’s horn	(Kurbanoglu and Algur, 2002)
*Bacillus licheniformis*	Submerged fermentation	Potato starch waste	(Liu et al., 2014)
*Rhodococcus opacus DSM 1069 and Rhodococcus opacus PD630*	Fermentation	Orange peel, lemon peel, corn husk	(Mahan et al., 2018)
**Microalgae**			
*Aphanothece microscopica Nägeli Chlorella sp. Fermentação submersa Meio de cultura padrão Zepka, 2010*	Submerged fermentation	Cheese whey, tofu whey and tempeh	(Putri et al., 2018)
*Chlorella sorokiniana*	Submerged fermentation	Rice bran	(Pruksasri et al., 2019)
*Galdieria sulphuraria*	Submerged fermentation	Culture medium	(Montenegro-Herrera et al., 2022)
*Dunaliella salina*	Submerged fermentation	Culture medium	(Sui et al., 2020)

*Saccharomyces cerevisiae* is a well-studied microorganism and its biotechnological utility lies in its unique biological characteristics, such as fermentation capacity and resilience to adverse conditions including low pH and osmolarity. In addition, it can be easily cultivated which allows for the maintenance of cell lines at low cost, and it is easily susceptible to genetic manipulation, allowing for both the addition and deletion of genes through a multitude of techniques (Parapouli et al., 2020; Munialo et al., 2022).

Current literature presents several studies on alternative proteins by fermentation using *S. cerevisiae* (Bacha et al., 2011; Aggelopoulos et al., 2014; Hezarjaribi et al., 2016; Akalya et al., 2017; Aruna et al., 2017; Gervasi et al., 2018; Bertasini et al., 2022). This yeast is very interesting because it meets the specifications necessary for the production of SCP, such as fast growth, low nutritional requirements, easy processing system, non-pathogenic and non-toxin production. In addition, yeasts are a good option for producing proteins of high biological value (Gervasi et al., 2018), with *Saccharomyces* being ranked among the most interesting due to its high protein content, which can reach more than 50% (Ziino et al., 1999). The yield and composition of the biomass formed is dependent on the process conditions and substrates used. Gervasi et al. (2018) and Aggelopoulos et al. (2014) found similar yields of 39.8 and 38.5%, respectively. In the studies by Hezarjaribi et al. (2016), on the other hand, a yield of 44.6% was verified, while Bacha et al. (2011) observed a value of 49.29%. Despite the diverse groups of microorganisms used as sources of SCP, yeasts from breweries are a globally accepted species, widely used in human food in a number of different countries (Anupamaa and Ravindra, 2000).

From the data obtained in WoS it was possible to observe that the genus *Candida* is the second most used for SCP production being the species *Candida utilis* and *Candida tropicalis* the most employed, followed in smaller proportion by the species *Candida blankii*, *Candida pseudotropicalis*, *Candida langeronii*, *Candida strains*, *Candida parapsilosis*, *Candida guilliermondii* among others. Work with *Candida* for SCP production can be seen in the studies by Ibrahim et al. (2004), Valentino et al. (2015), Jinru Jia et al. (2017), Kurcz et al. (2018), Mora et al. (2019) and Carranza-Méndez et al. (2022). Carranza-Méndez et al. (2022), when used *Candida utilis* for SCP production from orange peel waste, described that this yeast is considered safe and has been used for quite some time in the food area in the production of various industrial products for human and animal consumption, and can consume various types of substrates, such as food waste, for SCP production. Regarding the *Saccharomycetaceae* family, the genus *Kluveromyces* is commonly used, with emphasis on the species *Kluyveromyces marxianus*, *Kluyveromyces fragilis*, *Kluyveromices cicerisporus* and *Kluyveromyces lactis*. Some of these species can be visualized on the keyword map (Figure 7). According to Ritala et al. (2017), the species *Kluyveromyces marxianus*, produces protein in interesting concentration with a yield of 43%. Karim et al. (2020) in turn, describes that this species is one of the most promising unconventional yeasts that has fast microbial growth rates, ability to utilize different substrates, as well as having thermotolerance and metabolites that can be specific for use in the food industry.

Among the group of filamentous fungi, the most widely used microorganisms are from the genus *Aspergillus*, where the species *Aspergillus niger* and *Aspergillus Oryzae* are the most common, being also observed studies with the species *Aspergillus awamori* var. *Aspergillus Kawachi* and *Aspergillus parasiticus*. The *Aspergillus niger* species can be visualized on the keyword map (Figure 7) and according to the literature study by Ritala et al. (2017), this species produces proteins with a yield of 17–20% and can be isolated for SCP production.

In the group of bacteria, the predominant genus is *Bacillus*, such as the species *Bacillus subtilis*, *Bacillus coagulans*, *Bacillus lichenformis*, *Bacillus stearothermophilus*, *Bacillus thuringiensis*, *Bacillus brevis*, and *Bacillus firmus*. In the sequence are the microalgae, such as *Chlorella pyrenoidosa*, *Thalassiosira weissflogii*, *Selenarstrum capricornutum*, *Scenedesmus* sp. *Scenedesmus dimorphus* and *Spirulina patensis* (Amorim et al., 2021). The species mentioned above were obtained from the data extracted from Web of Science for the survey of this research according to the methodology applied.

In addition to the incredible variety of microorganisms used in this process, the production of SCP is further diversified by the types of fermentation which may be utilized Table 4. In the keyword map, the terms, “solid state fermentation” and “submerged fermentation” were observed, and these processes are widely used. Solid state fermentation is a process that requires substrates in solid form with adequate moisture level between 60–65% (Sharif et al., 2021). This technique has been practiced for centuries in the East and in Asian countries *via* applications in the food sector such as the production of fermented foods such as soybeans and rice (Manan and Webb, 2017).

Solid state fermentation is being commonly used for the production of SCP (Ravinder et al., 2003; Jaganmohan et al., 2013; Aruna et al., 2017) and according to Antoine et al. (2010), it has gained interest due to a number of economic and engineering advantages, including simplicity of equipment, low moisture content (which avoids bacterial contamination), low energy consumption, minimization of problems caused by low gas distribution, and differential expression of metabolites. Among the articles extracted from WoS, it was observed that for fermentative process cited, the most used microorganisms are from the genus *Saccharomyces*, *Aspergillus*, *Candida* and *Bacillus.*

In submerged fermentation, on the other hand, the microorganism is inoculated into a liquid medium containing all the nutrients necessary for its growth, and this process is carried out in a fermenter (Hezarjaribi et al., 2016; Oshoma and Eguakun-Owie, 2018; Ahlborn et al., 2019; Salgado et al., 2021; Sharif et al., 2021). According to the search on the WoS platform, the genera of the microorganisms most employed in this type of fermentation are *Saccharomyces*, followed by *Candida*, *Kluyveromyces*, and *Aspergillus*. Among the advantages of submerged fermentation are the ease of handling and better process monitoring when compared to solid state fermentation. This type of fermentation facilitates the aeration process that allows for a greater increase in oxygen and favors the oxidation of substrates (Reihani and Khosravi-Darani, 2019). Furthermore, it allows for the more rapid production of mycelial biomass (Strong et al., 2022). It is because of these and other advantages that this fermentative process is mostly used in articles involving SCP, as noted in the WoS searches of the investigated articles. However, the high cost of production, low productivity and the complexity of the medium are the main disadvantages associated with submerged fermentation (Manan and Webb, 2017).

The composition of the culture medium has considerable effects on the rate of cell growth and consequently on the production of biomass (Ahmadi et al., 2010; Hezarjaribi et al., 2016) The literature shows a variety of substrates that can be used (Table 4), and new research has been carried out in the search for new sources of nutrients. In the bibliometric map, words related to agro-industrial residues were observed, such as cheese whey, whey, apple pomace, wastewater, rice bran, among others. It is known that SCP for human consumption is usually produced from food-grade substrates (Ritala et al., 2017), however, yeast extract as a source of nutrients for the industrial growth of microorganisms is limited by the market price (Duarte et al., 2008).

Thus, a practice that has been trialled is the use of by-products from food and beverage processing industries, as well as forestry and agricultural sources, for the production of SCP as an economic and environmental strategy to reduce costs and reuse waste. (Ritala et al., 2017; Rakicka-Pustułka et al., 2021). The use of agro-industrial residues is further of interest because these sources provide the carbon and nitrogen necessary for the growth of microorganisms (Anupamaa and Ravindra, 2000).

An interesting survey was recently released by the Good Food Institute (GFI) which reported the launch of Brave Robot ice cream by the company Perfect Day in the year 2021.This product is a whey protein ice cream produced *via* the fermentation process of the microflora of fungi grown in tanks (Reference GFI website). Another attractive proposal has been the fermentation of agro-industrial residues for the production of animal feed with higher protein content (Yunus et al., 2017; Zhou et al., 2017; Patsios et al., 2020; Uwineza et al., 2021; Vethathirri et al., 2021).

Another recurring term in the network map is “optimization” which also appears among the most cited keywords. It is known that process conditions such as type of culture, strain improvement, type and application of substrate pre-treatments, nutrient supplementation, types of fermentation and environmental conditions such as incubation temperature, pH, dissolved oxygen and aeration can alter the biological processes that lead to the production of SCP (Anupamaa and Ravindra, 2000; Hezarjaribi et al., 2016).

Thus, several researchers (Ghaly and Ben-Hassan, 1995; Hezarjaribi et al., 2016; Ji et al., 2016) have studied different process conditions in order to optimize SCP production. Kamal et al. (2019) for example, studied the influence of temperature, pH, substrate concentration and fermentation period on protein yield. Umesh et al. (2019) on the other hand, evaluated the variables temperature, pH, incubation period, carbon and nitrogen sources.

The results described above show that bibliometric analysis has been a useful tool for disseminating best practice, as well as documenting and synthesizing general trends in this field of knowledge, highlighting the scientific importance of studies of this nature.

### 3.4. Future challenges and trends

SCP technology is an interesting alternative to animal protein whose potential sustainability may help solve environmental problems and population growth. Ahmad et al. (2022) reported that SCP has the potential to replace traditional meat as well as plant-based protein alternatives. However, the safety of these products is a consideration which must be addressed. Some concerns related to the consumption of SCP have been discussed by the scientific community and many efforts have been made in order to guarantee the quality of the product as well as the health of the consumer.

The production of microbial toxins is one of the main concerns associated with SCP. These compounds are secondary chemical species arising from the metabolism of microorganisms that threaten food security, economies and can cause negative health effects. According to Hashempour-Baltork et al. (2020), these metabolites are capable of causing allergic reactions in consumers, as well as potential nephrotoxic, immunosuppressive, carcinogenic and teratogenic effects (Pfohl-Leszkowicz and Manderville, 2007; Marin-Kuan et al., 2011; Hoeltz et al., 2012). Some species have the ability to inhibit protein synthesis such as peptide transferase activity Stafford and Mclaughlin (1973), in addition, they contribute to thymic aplasia, potential liver and hepatocellular cancer, and have oncogenic and immunosuppressive properties (Raisuddin, 1993; Groopman et al., 2008; Da Rocha et al., 2014). Therefore, many studies have discussed the need for research on food safety and quality control in the production of SCP.

A variety of analytical techniques can be used during product processing. Accordingly, one proposal is the decontamination of the reaction medium by the fungi present in the production process, eliminating the toxins through a phenomenon called bioremoval (Ji et al., 2016; Venkatesh and Keller, 2019). The reduction of mycotoxins during the fermentation process can occur *via* the adsorption of these substances by the cells of the microorganisms, as well as by the production of enzymes responsible for the degradation of these contaminants or the removal of toxin-generating genes through genetic improvement (Adebiyi et al., 2019). A successful example of a SCP product with excellent quality standards is the Quorn brand mycoprotein which uses the fungus *Fusarium venenatum* in the fermentation process and was considered safe by the US Food and Drug Administration (Hashempour-Baltork et al., 2020; Ahmad et al., 2022).

Another challenge to the adoption of this technology is the mass production of RNA during the fermentation process. The production of RNA which occurs during this process can be problematic because this by-product acts as an anti-nutritional factor in the final product (Sharif et al., 2021). An interesting strategy to address this problem is the application of heat treatment, a technique used in Quorn brand products, where the protein biomass is subjected to a temperature of 72–74°C for 30–45 min to reduce the RNA content to an acceptable level for human consumption (Ahmad et al., 2022).

Another potential challenge that has been discussed is the use of microalgae for the production of SCP. Some types of microalgae, such as Spirulina, Chlorella, Scenedesmus, are already widely used (Oliveira and Leite, 1999; Li et al., 2013; Duong et al., 2015). The protein products of these microalgae can reach up to 70% fatty acids, mineral salts, vitamins, antioxidant activity, as well as interesting process conditions such as rapid growth, simple cultivation and good use of solar energy (Sharif et al., 2021) However, there are some limitations to the use of these microorganisms for SCP production.

Microalgae have in their cell wall, cellulose that cannot be digested by humans (Ghasemi et al., 2011). One strategy used by food companies has been the disruption of cell walls during product processing through mechanical or non-mechanical/chemical methods. Among the mechanical processes are the use of mechanical mills, homogenizers and ultrasound devices. Among the non-mechanical methodologies are microwave, pulsed electric field, enzymatic action, ionic liquid, organic solvents, extraction with critical and supercritical fluids, and other chemical products (Waghmare et al., 2016; Amorim et al., 2021).

Another issue which can pose challenges to the use of microalgae for production, especially in open systems, is the possibility of climatic variations, contamination of the productive environment. This contamination may be biological in nature, or perhaps mineral, caused by the bioaccumulation of toxins and ions of heavy metals (Harun et al., 2010; Rzymski et al., 2015; Ritala et al., 2017). The presence of species of cadmium, mercury, lead, as well as phycocyanins and phycobiliproteins have been reported, which may indicate the development of allergic processes in consumption of microalgal material (Geh et al., 2015; Rzymski et al., 2015).

Another subject of interest is the use of genetic manipulation of microorganisms for the production of SCP. Several studies have worked with modified microorganisms for the production of SCP. In this scenario, Cernak et al. (2018) used the CRISPR-Cas9 technique in the yeast *Kluyveromyces marxianus* to impart the ability to produce lipids and acquire thermotolerance, opening perspectives for the use of this yeast on an industrial scale in food production.

The literature also presents other applications with the aim of achieving specific biotechnological goals (Karim et al., 2020). On such example is Srivastava et al. (2020) and Gupta et al. (2020) who, through genetic modifications in transcription factors, induced the overexpression of carbon transporters in cyanobacteria *Synechococcus* sp. to increase the growth rate and amount of single cell protein produced (Tramontin et al., 2019). In 2021, the Food and Drug Administration (FDA) approved for commercial use KnipBio Meal, a substitute for fish meal consisting of SCP produced by the yeast *Yarrowia lipolytica* genetically modified to produce high levels of astaxanthin, improving its nutritional content (Tlusty et al., 2017; Tramontin et al., 2019).

Genetic engineering can also be applied to the production of other nutritional and functional compounds that are not naturally expressed by microorganisms or to increase the production of nutrients already produced by manipulating biosynthetic pathways (Balagurunathan et al., 2022).

Despite the many benefits of genetic manipulation for food and feed applications, regulatory requirements on the integration of genetically manipulated products into food can be complex and vary widely in different jurisdictions. The use of modified microorganisms for the production of enzymes and other metabolites, in which case the organism is completely absent from the final product, is already commonplace. However, propositions where genetically modified microorganisms are constituents of the final product are more delicate. In such cases, regulations are more stringent and acceptance varies globally depending on the method and nature of the modification (Strong et al., 2022).

Another potential challenge in this area is the use of food waste and the like as alternative substrates to glucose for fermentation processes, such as lignocellulosic biomass that is abundant and does not have many utilization strategies (Wikandari et al., 2021). However, although the use of by-products derived from agro-industries for the production of SCP is an interesting proposal from the point of view of cost and sustainability, it is important to note that it is quite challenging from the position of safety and origin of the raw material. Food produced from waste needs to be accompanied by records that guarantee product safety and consumer health (Ritala et al., 2017).

Further concerns for SCP utilization revolve around the health aspects of mycoprotein consumption. There remain a number of questions in regard to the safety of human consumption of such products, and further improvements are necessary to address such lingering experimental questions as lipid effects, level of satiety, muscle protein synthesis, effects of large volumes of these macromolecules on the human body, and suggested daily dosing (Ahmad et al., 2022).

## 4. Concluding remarks

The present study carried out a scientific mapping of the composition and structure of the realm of alternative protein production by fermentation processes. In doing so it provided an overview of the main themes, publications and patent deposits, as well as contributors, journals and main keywords which may aid investigators interested in this line of research. This compilation of research revealed that SCP technology has been well-studied with significant increases in the number of publications and patent filings in the last decade. It further emphasized the interdisciplinary nature of the subject, highlighting that many organizations are involved in the research, production and commercialization of this product, lending to a very promising and growing market. Overall, the potential sustainability of this technology makes it an interesting and appealing alternative to traditional proteins. However, it is important to emphasize that there remain gaps in knowledge and a number of challenges which have yet to been fully described. Therefore, further research and development will be necessary for the improved understanding and potential adoption of this technology.

## Data availability statement

The original contributions presented in the study are included in the article/supplementary material, further inquiries can be directed to the corresponding author.

## Author contributions

GR, LR, and TS contributed to the researching, graphics construction, and writing of this article. JA provided information on microbial toxins, microalgae, and writing the article. RO, TN, JB, and MS reviewed, edited, and translated the manuscript. All authors contributed to the article and approved the submitted version.

## Conflict of interest

The authors declare that the research was conducted in the absence of any commercial or financial relationships that could be construed as a potential conflict of interest.

## Publisher’s note

All claims expressed in this article are solely those of the authors and do not necessarily represent those of their affiliated organizations, or those of the publisher, the editors and the reviewers. Any product that may be evaluated in this article, or claim that may be made by its manufacturer, is not guaranteed or endorsed by the publisher.
